# The Prognostic Model Based on Tumor Cell Evolution Trajectory Reveals a Different Risk Group of Hepatocellular Carcinoma

**DOI:** 10.3389/fcell.2021.737723

**Published:** 2021-09-29

**Authors:** Haoren Wang, Shizhe Yu, Qiang Cai, Duo Ma, Lingpeng Yang, Jian Zhao, Long Jiang, Xinyi Zhang, Zhiyong Yu

**Affiliations:** ^1^Department of Hepatobiliary Surgery, The Affiliated Hospital of Yunnan University, Kunming, China; ^2^Department of Oncology, The First Affiliated Hospital of Zhengzhou University, Zhengzhou, China; ^3^Department of Surgery, The First Affiliated Hospital of Zhengzhou University, Zhengzhou, China

**Keywords:** hepatocellular carcinoma, single-cell transcriptomics, tumor heterogeneity, tumor evolution, copy number aberration, prognosis, cell state transition, genomic diversity

## Abstract

Hepatocellular carcinoma (HCC) is one of the leading causes of cancer-related death worldwide, and heterogeneity of HCC is the major barrier in improving patient outcome. To stratify HCC patients with different degrees of malignancy and provide precise treatment strategies, we reconstructed the tumor evolution trajectory with the help of scRNA-seq data and established a 30-gene prognostic model to identify the malignant state in HCC. Patients were divided into high-risk and low-risk groups. C-index and receiver operating characteristic (ROC) curve confirmed the excellent predictive value of this model. Downstream analysis revealed the underlying molecular and functional characteristics of this model, including significantly higher genomic instability and stronger proliferation/progression potential in the high-risk group. In summary, we established a novel prognostic model to overcome the barriers caused by HCC heterogeneity and provide the possibility of better clinical management for HCC patients to improve their survival outcomes.

## Introduction

Hepatocellular carcinoma (HCC), with more than one million new cases annually and a 5-year survival rate <20% in most countries, is the fastest growing malignancy both in terms of incidence and mortality (Allemani et al., 2018; Bray et al., 2018). Despite the clinical efficacy of systemic therapies such as tyrosine kinase inhibitors (TKIs) and immune checkpoint inhibitors (ICIs), primary and secondary drug resistance is inevitable and ultimately leads to treatment failure (Llovet et al., 2008, 2018; Abou-Alfa et al., 2018; Pinter et al., 2021). This ability of HCC to adapt to pharmacologic pressures can be described as tumor evolution and can be attributed to the heterogeneity of HCC (Amirouchene-Angelozzi et al., 2017), which refers to the different genetic or epigenetic alterations within the same lesion (intratumor heterogeneity) or in different lesions in the same patient (inter-tumor heterogeneity). Thus, the understanding of the potential mechanisms underlying HCC heterogeneity and its impact on therapeutic intervention is paramount for treatment success and overall survival (OS; Llovet et al., 2021).

The traditional bulk RNA-seq only provides the average number of genes expressed in a pooled population of cells and cannot detect the wide transcriptome heterogeneity in cell populations (Wang et al., 2020). Thus, researchers previously classified cells by purpose-related features and focused on a set of genes, ignoring the continuity of the tumor evolution process (Bidkhori et al., 2018; Chaudhary et al., 2018; Long et al., 2019b; Sarathi and Palaniappan, 2019; Sun et al., 2020; Zhang et al., 2020; Zhu et al., 2020). However, with the advent of single-cell RNA sequencing (scRNA-seq), a novel technology that allows transcriptomic analyses of individual cells, researchers can explore the heterogeneity and plasticity of tumor cells, which can result in early recurrence and drug resistance in the process of tumor evolution on single cell resolution. Given the large number of cells, we can reasonably hypothesize that the sequencing results include every distinct point of the dynamic process (Losic et al., 2020; Marjanovic et al., 2020; Rajewsky et al., 2020; Sun et al., 2021). Several studies have profiled the single-cell landscape of tumor generation and progression (Kim et al., 2018; Durante et al., 2020; Losic et al., 2020; Marjanovic et al., 2020). Furthermore, scRNA-seq is a promising tool that can facilitate individualized therapy owing to its ability to define cell subsets with potential treatment targets.

Here, we reconstructed the evolution trajectory of tumor cells with the help of scRNA-seq data and established a prognostic model to classify different risk groups of HCC. Our findings provide a strategy for precision medicine on the basis of tumor heterogeneity, and we also identified a wide range of potential therapeutic targets, thus improving the survival of patients with HCC.

## Materials and Methods

### Data Sources

The normalized gene-level RNA-Seq data and clinical information for 364 patients LIHC-TCGA cohorts were downloaded from UCSC Xena^1^ with R package UCSC Xena Tools (Wang and Liu, 2019). To obtain 258 patients LIRI-JP validation set, RNA-seq data, and related clinic pathological data were downloaded from the ICGC website^2^ (Zhang J. et al., 2019). The scRNA-seq barcode sequences and raw gene expression matrix were downloaded from the CNP0000650 (Sun et al., 2021). Mutation data that contained somatic variants were stored in Mutation Annotation Format (MAF) form and were downloaded from Genomic Data Commons (GDC).^3^

### Processing of Single-Cell RNA-Seq Data

#### Dimension Reduction and Unsupervised Clustering

Single-cell RNA sequencing data were processed for dimension reduction and unsupervised clustering by following the workflow in Seurat (v4.0.2) (Butler et al., 2018). In brief, first, the read counts for each cell were divided by the total counts for that cell and multiplied by the scale factor (10,000), and then natural-log transformed. A principal component analysis (PCA) matrix with 50 components were calculated to reveal the main axes of variation and the data were denoised by using “Run PCA” function with default parameter. For visualization, the dimensionality of each dataset was further reduced using Uniform Manifold Approximation and Projection (UMAP) implemented in “Run UMAP” function (Becht et al., 2019). We retained cell clustering based on the original study. The cluster-specific marker genes were identified by using the “Find All Markers” function with MAST algorithm (Finak et al., 2015).

#### Define Subpopulations of Aneuploid Tumor Cells

TPM gene expression matrix was extracted from the Seurat object as recommended in the “prepare the read count input file” section (CopyKAT). For each patient, normal reference T cells and malignant cells were selected and identified from the annotated clusters as determined above. Quality control filtering was performed to select the highest quality cells by only including malignant cells with at least five genes in each chromosome to calculate DNA copy numbers. We extracted aneuploid cells that are considered as tumor cells in aneuploid tumors to define two copy number subpopulations of single tumor cells using default parameters in CopyKAT (Gao et al., 2021).

#### Construct Tumor Cell Evolution Trajectory

Malignant cells were identified from the annotated clusters as determined above. This resulted in six high-quality malignant clusters to use for this analysis. Single-cell pseudo-time trajectories were constructed with Monocle 2 (2.10.1) (Qiu et al., 2017). Genes for trajectory inference were selected using the “dispersion table” function to calculate a smooth function describing how variance in each gene’s expression across cells varies according to the mean. Only genes with mean expression greater than or equal to 0.1 were used for the analysis. The “reduce Dimension” function was utilized with the DDRTree reduction method with default parameters. Results were visualized using the “plot cell trajectory” and “plot complex cell trajectory” functions and annotated with cell type, subclones, and calculated cell states. Once the pseudo-space trajectory was defined, we used the Tradeseq (Trajectory Differential Expression analysis for Sequencing data) R package to select genes that were differentially expressed along the trajectory (Van den Berge et al., 2020). Association Test function was used to test whether the average gene expression is significantly changing along pseudotime. The top 500 gene upregulated genes and Top 500 downregulated genes decrease along the inferred pseudo-time trajectory with a *q*-value less than 0.01 were separated with hierarchical clustering using the “plot multiple ranches heatmap” function with num clusters = 3 and “branches” set to the terminal branchpoints for aneuploid tumor cells.

#### Development and Validation of the Tumor Evolution Signature for Hepatocellular Carcinoma

Select differential genes based on single cell tumor evolution trajectory to reduce the impact of non-tumor cells. The cases from the TCGA LIHC datasets were used as the training cohort to establish the LASSO model. Univariate analysis and logRank test were used to identify genes with prognostic ability. For the genes with prognostic ability, Cox proportional hazards model (iteration = 1,000) with a lasso penalty was used to find the best gene model utilizing an R package called “glmnet” (Friedman et al., 2010). The best gene model was used to establish the tumor evolution signature. The risk score for each patient was calculated with the LASSO model weighting coefficient as follows:


$$r\,i\,s\,k\,s\,c\,o\,r\,e\,s=\sum_{i=1}^{n}C\,o\,e\,f_{j}*X_{j}$$

In this formula, *n* represents the number of key genes, *Coef*_*j*_ is the LASSO coefficient of Gene *j*, and *X*_*j*_ is the normalized expression value of Gene *j* (Supplementary Table 4). Then, the concordance (c)-index proposed by Harrell^24^ was applied to validate the predictive ability of the signature in all datasets, by using the “survcomp” R package (Haibe-Kains et al., 2008). The larger c-index indicated the more accurate predictive ability of the model.

### Survival Analysis

To verify the trend of this tumor evolution trajectory, Kaplan--Meier (K--M) analysis was performed. The top 10 end-genes were extracted and the potential prognostic significance of these genes was assessed with the LIHC data from GEPIA2.^4^ The K–M survival curves were also generated to graphically demonstrate the OS to the high-risk group and low-risk group, which were stratified by the tumor evolution signature. The R package called “survminer” was utilized to perform the survival analysis, and the optimal cutoff was ascertained by the “surv_cutpoint” function.

### Somatic Mutation and Copy-Number Aberration Analysis

Mutation comment file (MAF) of TCGA-LIHC cohort was downloaded from the GDC client. Differential analysis and visualization of somatic mutations was done using Maftools (The Cancer Genome Atlas Research Network et al., 2013; Mayakonda et al., 2018). This difference between high- and low-risk group was detected using function “mafComapre,” which performs Fisher’s exact test on all genes between two groups to detect differentially mutated genes.

Composite copy number profiles were generated to highlight differences between high- and low- risk group. Segment file of TCGA-LIHC cohort was downloaded from FIREHOSE and samples were further divided into high- and low-risk groups. Then we ran the GISTIC 2.0 pipeline to generate discrete copy number data file. Chromosomes reference objects were from the “BSgenome.Hsapiens.UCSC.hg19” R package.

As in the previous study, a non-synonymous mutation from the TCGA database was used as the raw mutation count, and it was divided by 38 MB to quantify TMB (Chalmers et al., 2017). The samples were sorted according to the value of the median TMB from low to high.

### Bioinformatics Analyses

Gene Enrichment Analysis (GSEA) was further used to investigate the functional enrichment of risk score-associated genes using the R package “clusterProfiler” (Yu et al., 2012). The Benjamini–Hochberg method was used to adjust nominal *p*-values (false discovery rate, FDR) for multiple testing.

The Maftools package was used to illustrate the respective mutation profiling of the two risk group levels by waterfall plot, and differentially mutated genes were identified by using the “mafCompare” function where genes mutated in greater than 5% of LIHC samples in the TCGA cohort were considered (Mayakonda et al., 2018).

### Statistical Analysis

Student’s *t-test* was conducted to make statistical comparison. The “pheatmap” R package was applied to generate heatmaps. Survival analysis was completed using Kaplan–Meier method, and the prediction performance of the risk model was evaluated using receiver operating characteristic (ROC) *via* “time-ROC” R package. Multivariate COX regression analyses were used to investigate the prognostic value of risk-score. Hazard ratio (HR) and 95% confidence intervals (CI) for each variable were also calculated where needed. A value of *p* < 0.05 was defined as statistically significant difference. All of our analyses were conducted using R software version 4.0.2.^5^

**FIGURE 1:**
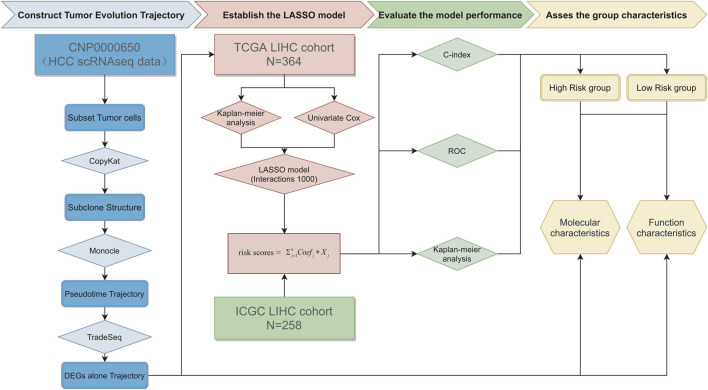
The whole process of data analysis.

The whole process of data analysis is depicted in Figure 1.

## Results

### Classification of the Malignant Cell Clusters With Single-Cell RNA Sequencing Data

**FIGURE 2:**
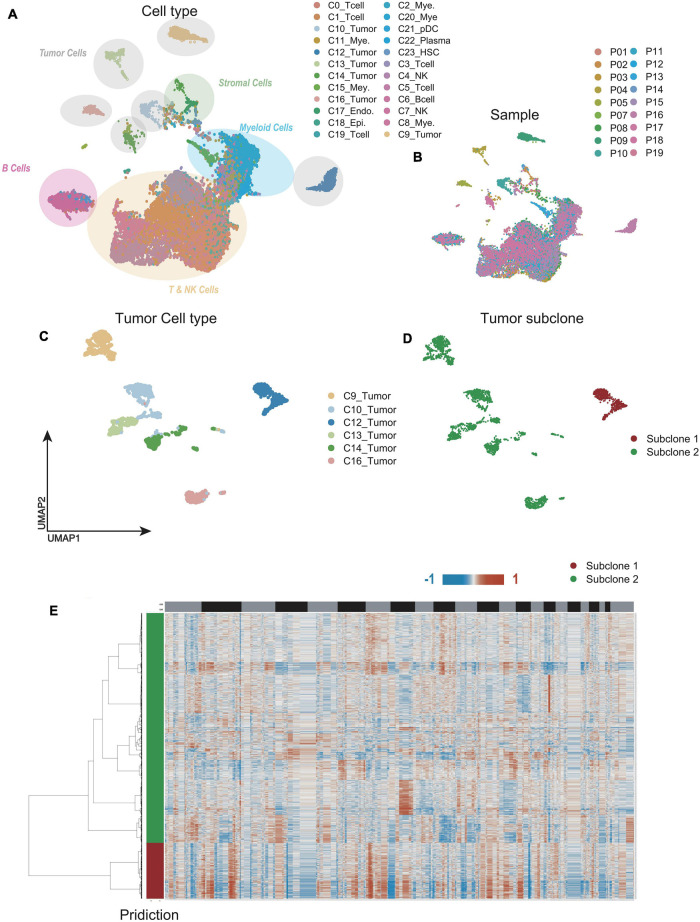
Single-cell RNA sequencing (scRNA-seq) profiling of different malignant cell clusters. **(A,B)** The Uniform Manifold Approximation and Projection (UMAP) plot showing the annotation and color codes for cell types in hepatocellular carcinoma (HCC) **(A)**. Cells were further shown in different color by patient origin **(B)**. **(C)** The UMAP plot, showing only malignant cell clusters by Louvain algorithm. **(D)** With CopyKAT, malignant cell clusters were delineated into two subclones by single-cell copy number profiles inferred from scRNA-seq data. **(E)** Clustered heat maps of single HCC malignant cell copy number profiles in two major subclones.

Unsupervised dimensionality reduction and graph-based clustering analysis were performed with the data from CNP0000650, and 24 clusters (Figure 2A) were visualized by the UMAP method (Becht et al., 2019; Sun et al., 2021). The immune cells mainly consisted of myeloid-derived cells, T cells, B cells, plasma cells, and natural killer (NK) cells, while non-immune cells included endothelial cells, hepatic stellate cells, apparently normal epithelial cells, and HCC malignant cells. To contrast the difference among different patients, we classified the cells by patient origin (Figure 2B), and the result showed that tumor cells contained obvious heterogeneity, while non-tumor cells kept homogeneous, proving that the differences between tumor cell clusters are mainly due to the tumor heterogeneity, rather than batch effects between samples. No normal liver cells were detected, likely because of the technical limitations, resulting in no comparison between normal and malignant liver cells.

We further extracted the varied tumor cell clusters for the analysis of tumor heterogeneity (Figure 2C), and two major sub-clones were defined by the clustered heat maps of single cell copy number profiles (Figure 2D). Compared with sub-clone 2 (green), the heatmap showed that sub-clone 1 (red) contained more CNAs, implying that sub-clone 1 might be more malignant (Figure 2E).

### Reconstruction of Tumor Cells Progression Trajectory

**FIGURE 3:**
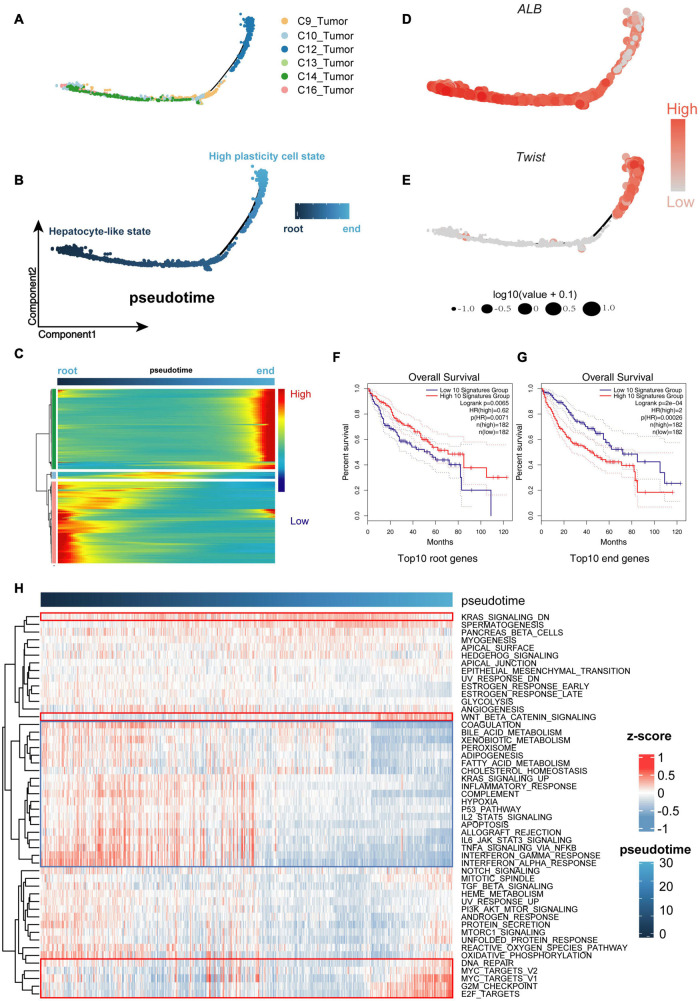
Cells were sorted by progression from lower malignant state to higher malignant state. **(A,B)** Cell Trajectory performing the route of low- to high-malignant cells, which can serve as a model to describe malignant cell differences. **(C)** Expression levels for differentially expressed genes (rows), with cells (columns) shown in pseudo-time order. **(D,E)** ALB and Twist genes confirming the trusty of the cell progression trajectory model. **(F,G)** The Kaplan–Meier (K-M) analysis of the top 10 downregulated genes (root genes) and top 10 upregulated genes (end genes) from 1,000 differential genes group capturing the overall survival (OS) differences between low- and high-malignant cell groups. **(H)** Gene set variation analysis (GSVA) heat map showing the mainly differential signaling pathways between low- and high-malignant cell groups.

To determine the relationship between malignant cell clusters, we performed single-cell trajectory analysis with scRNA-seq data using Monocle (Qiu et al., 2017). As is well known, genomic mutations accumulate over time in the process of tumor evolution (Turajlic et al., 2019; Craig et al., 2020; Losic et al., 2020). Thus, we defined the cell cluster with a lower CNA burden as the root, while the cell cluster with a higher CNA burden was defined as the end of the trajectory. We noticed that sub-clone 2, comprising cells with obvious liver characteristics, was concentrated at the beginning of the trajectory, while sub-clone 1, comprising cells with less specificity of origins, was concentrated at the end of the trajectory, indicating that the trajectory model fits the process of tumor evolution well (Figures 3A,B).

During the process of transition of tumor cells from a lower to higher malignant state, some genes are silenced, while others become newly active. We used Tradeseq, a powerful generalized additive model framework based on the negative binomial distribution, to interpret the within lineage differential expression (Figure 3C and Supplementary Table 1). To verify the malignant trend of this trajectory, we extracted the top 10 downregulated genes (root genes) and top 10 upregulated genes (end genes) for Kaplan–Meier (K-M) analysis. The high expression of the top 10 root genes represented a benign prognosis, while the high expression of the top 10 end genes represented a poor prognosis (Figures 3F,G). Furthermore, along the trajectory, liver characteristics such as *ALB* (Figure 3D) were gradually lost, while stemness and malignant marker genes such as *TWIST1* (Figure 3E), gradually increased, suggesting that the malignant state was advancing.

The gene set variation analysis (GSVA; Hänzelmann et al., 2013) was used to further analyze the underlying biological processes along the trajectory. In the Molecular Signature Database (MSigDB) “hallmark” collection of major biological categories (Liberzon et al., 2015), the upregulated genes of sub-clone 1 were enriched in the tumor-promoting pathway (“Wnt/β-catenin signaling”) and proliferation pathway (“G2M checkpoint,” “E2F Targets,” “MYC Targets”), while the downregulated genes of sub-clone 1 were enriched in the tumor-suppressor pathway (“P53 pathway”) and essential liver function pathway (“Complement,” “Fatty acid metabolism,” “Adipogenesis”) (Figure 3H), which were consistent with the characteristics of cells that progressed from the lower malignant state to the higher malignant state (Maley et al., 2017; Losic et al., 2020; Marjanovic et al., 2020; Llovet et al., 2021).

### Establishment of the 30-Gene Prognostic Model

**FIGURE 4:**
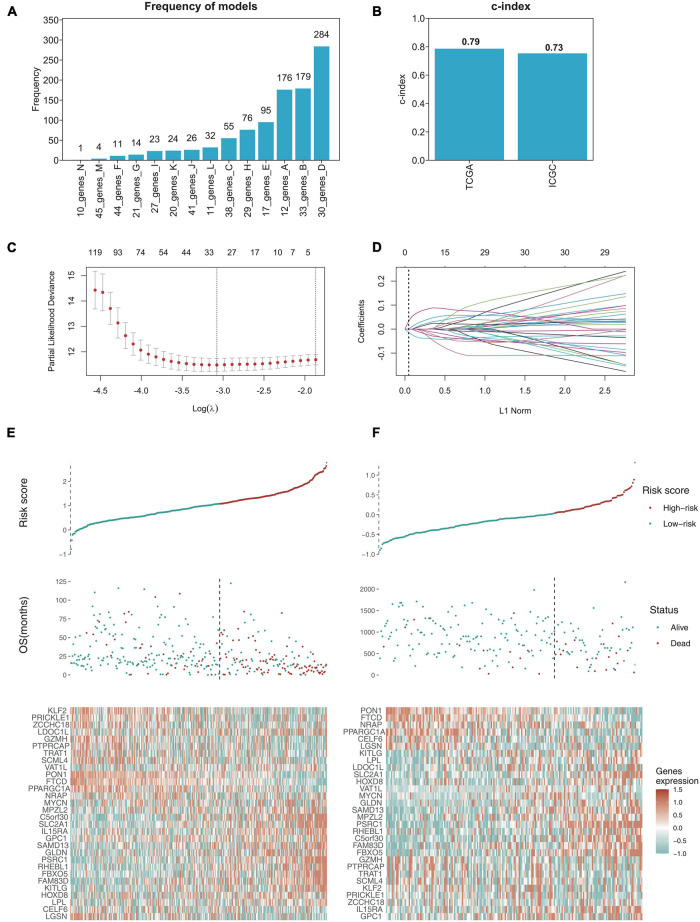
Establishing the 30-gene prognostic model with LASSO regression analysis. **(A)** LASSO regression analysis performed the frequency of different gene combination models and finally determined the 30-gene signature for OS prediction. **(B)** C-index of 30-gene prognostic model was 0.79 in TCGA training cohort, while 0.73 in ICGC validation cohort. **(C)** LASSO coefficient profiles of the gene features. **(D)** Ten-time cross-validation for tuning parameter selection in the LASSO model. **(E,F)** The risk score distribution and survival status distribution of 30-gene prognostic model in TCGA training cohort and ICGC validation cohort, and the heat map of gene expression are shown below with color, red (high) and green (low).

Although the top 10 genes had a certain predictive effect, we preferred optimizing gene combination to obtain a better prognostic model. With the selection criteria of *p* < 0.01, the intersection of univariate Cox regression analysis and K-M analysis identified 200 credibly survival-related genes. We used TCGA data as the training cohort and ICGC data as the external validation cohort (The Cancer Genome Atlas Research Network et al., 2013; Zhang J. et al., 2019). Lasso-penalized Cox analysis was subsequently performed 1,000 times in the TCGA training cohort with 10-fold cross-validation to evaluate and eliminate variables that contributed less to the model, and a 30-gene signature with the most powerful predictive features were selected (Figures 4A,C,D). To validate the credibility of this model, C-index was assessed in the TCGA training cohort and ICGC validation cohort, which was confirmed as 0.79 and 0.73, respectively (Figure 4B), suggesting that our model had favorable efficacy for predicting prognosis (Harrell, 1982). Based on the 30-gene prognostic model, TCGA and ICGC samples were clustered into high-risk and low-risk groups, and the OS time of patients in the high-risk group was remarkably decreased (Figures 4E,F).

### Evaluation of the Prognostic Model in TCGA Cohort and ICGC Cohort

**FIGURE 5:**
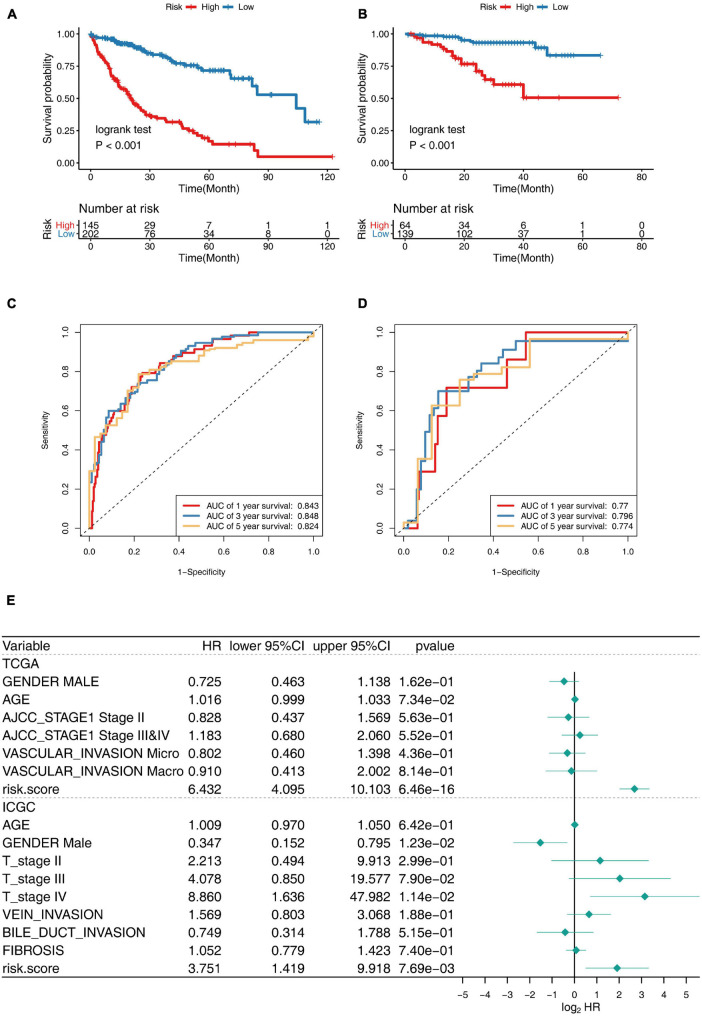
Prognostic performance of 30-gene signature in TCGA Training Cohort and ICGC Validation Cohort. **(A,B)** K–M survival curve for risk score in TCGA training cohort **(A)** and ICGC validation cohort **(B)**. **(C,D)** Receiver operating characteristic (ROC) curve of the 30-gene prognostic model in TCGA cohort **(C)** and ICGC cohort **(D)**. **(E)** Multivariate Cox regression analysis of clinical parameters and prognostic model for OS.

The K–M analysis and time-dependent ROC was used to assess the prognostic capacity of the 30-gene prognostic model in the TCGA cohort and ICGC cohort, respectively. The K–M analysis illustrated that patients in the low-risk group had significantly longer OS than those in the high-risk group, both in the TCGA cohort (Figure 5A) and the ICGC cohort (Figure 5B). The area under the ROC curve (AUC) for the 1-, 3-, and 5-year OS was 0.843, 0.848, and 0.824 in the TCGA cohort, while it was 0.77, 0.796, and 0.774 in the ICGC cohort (Figures 5C,D), indicating that this 30-gene prognostic model had high sensitivity and specificity for survival prediction.

Gender, age, stage, vascular invasion, bile duct invasion, fibrosis, and the risk score of the prognostic model were included in the multivariate Cox regression model, and the risk score was revealed to be independent predictor for OS, showing splendid predictive performance ability, with HR: 6.432, 95% CI: 4.095–10.103, *p* < 0.001 in TCGA cohort, and HR: 3.751, 95% CI: 1.419–9.918, *p* < 0.001 in ICGC cohort. Taken together, the 30-gene prognostic model was completely reliable for the precise prediction of OS in HCC (Figure 5E).

### Comparison of Genomic Aberrations in Different Risk Groups

**FIGURE 6:**
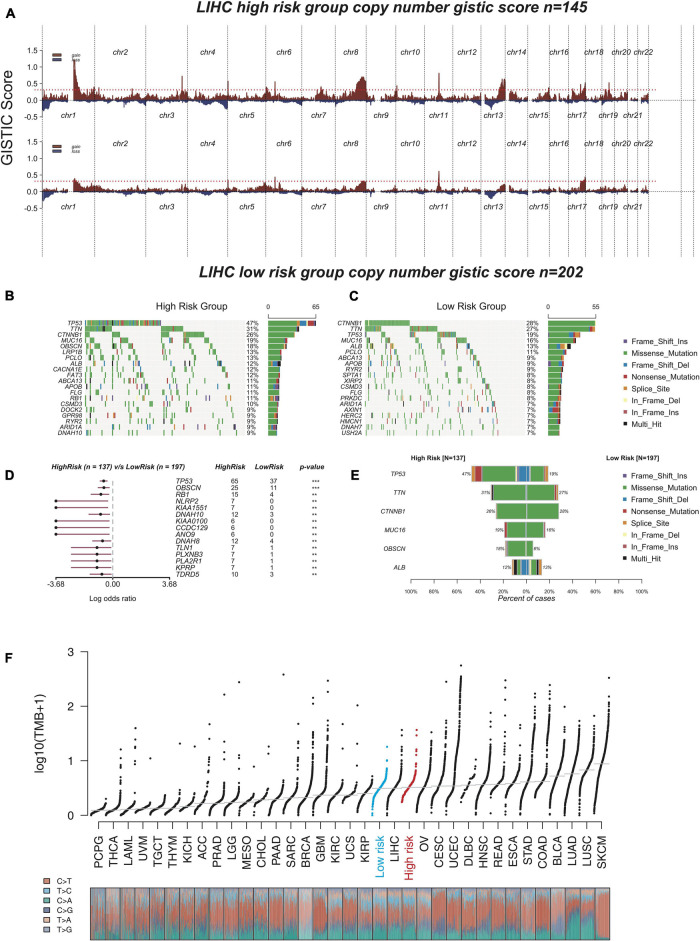
The analysis of genomic aberrations in high-risk group and low-risk group. **(A)** Recurrent copy number aberrations of high-risk group and low-risk group in TCGA cohort. Regions of recurrent copy number amplifications (red) and deletions (blue) were above and below baseline (0.0), respectively, in the targeted array were identified by GISTIC 2.0. (Red line represented GISTIC score of 0.3). **(B,C)** Oncoplot displaying the somatic landscape of high-risk group **(B)** and low-risk group **(C)**. Genes were arranged according to their mutation frequency. The *Y*-axis was the gene name and the abscissa was the sample name. Different colors represented different mutation types. **(D)** Forest plot showed differentially mutated genes between high-risk group and low-risk group. The adjacent table included the number of samples in high-risk group and low-risk group with the mutations in the highlighted gene. The *p*-value indicated significance threshold: ***p < 0.001; **p < 0.01; Fisher’s exact test. **(E)** Co-bar plots showed the most recurrently mutated genes in high-risk group and low-risk group. **(F)** The distribution plot shows tumor mutation burden (TMB) distribution of different cancer types. Liver hepatocellular carcinoma (LIHC) patients were divided into low-risk group and high-risk group.

Increasing mutation frequency is a typical feature of human cancer. To identify the divergence of genomic aberrations between the high-risk and low-risk groups in the TCGA database, CNAs data were downloaded from the GDC portal and analyzed with GISTIC 2.0, with which the high-risk group had an obviously higher genomic aberration burden than the low-risk group (Figure 6A). Meanwhile, the top 20 genes with high genomic mutation frequency in the high-risk and low-risk groups were constructed by Maftools (Mayakonda et al., 2018; Figures 6B,C). To analyze the discrepancy between the high-risk and low-risk groups, the differentially mutated gene type and frequency were compared by Fisher’s exact tests. The results showed three significantly differential genes—*TP53* (47 versus 19%), *OBSCN* (18 versus 5%), and *RB1* (11 versus 2%) (Figure 6D). The six genes most recurrently mutated were *TP53* (47 versus 19%), *TTN* (31 versus 27%), *CTNNB1* (26 versus 28%), *MUC16* (19 versus 16%), *OBSCN* (18 versus 5%), and *ALB* (12 versus 13%) (Figure 6E and Supplementary Table 2). These findings suggested that some mutated genes such as *TP53* and *OBSCN* that were notably different compared with the high-risk and low-risk groups could be related to the malignant progression and can continuously accumulate mutations over time; whereas, the others such as *CTNNB1* and *ALB*, which remained stable from the low-risk state to high-risk state, likely contribute to the essential neoplastic process rather than malignant progression.

The tumor mutation burden (TMB) is also considered an essential factor impacting on the occurrence and progression of the tumor. The distribution plot shows TMB distribution of different cancer types (Figure 6F). The three types including low-risk HCC, all HCC samples (liver hepatocellular carcinoma, LIHC), and high-risk HCC were apparently distinct from each other, and the TMB gradually increased from low-risk type to high-risk type, which suggested that our model had excellent distinguishing capability.

### Gene Enrichment Analysis of the 30-Gene Prognostic Model

**FIGURE 7:**
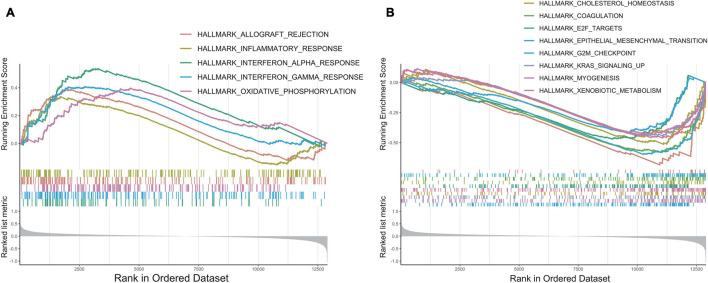
GSEA enrichment analysis. **(A)** The enrichment plot of upregulated gene sets in low-risk group. **(B)** The enrichment plot of downregulated gene sets in low-risk group.

To explore the underlying molecular mechanisms of this prognostic model, we conducted GSEA to compare the low-risk group with the high-risk group in TCGA cohorts (Yu et al., 2012). In the MsigDB “hallmark” collection of major biological categories, proliferative signaling pathways (“E2F targets,” “G2M checkpoint,” “KARS signaling”), and the invasion and metastasis-related signaling pathways (“EMT” and “myogenesis”) were dramatically increased in the high-risk group (Figure 7B). This was consistent with the data at the single-cell level. Notably, the low-risk group was enriched in inflammation-related gene pathways such as “inflammatory response,” “interferon-α response,” and “interferon-γ response,” which suggested that the secretion of inflammatory factors might originate from the tumor cells, indicating a strong proinflammation potential in the early tumorigenesis stage (Figure 7A). A similar phenomenon had been reported in melanoma, breast cancer, and colorectal cancer, where the expression of immune-related genes was also presented by tumor cells and could likely be an independent influence on their prognostic differences (Sconocchia et al., 2014; Forero et al., 2016; Buetow et al., 2019; McCaw et al., 2019; Jin et al., 2020).

## Discussion

Hepatocellular carcinoma is one of the leading causes of cancer-related mortality worldwide. Previous studies have proved that the heterogeneity, which was thought to be evolutionarily selected for increasing fitness of tumor cells, might be the major barrier for improving patients outcome (Chaudhary et al., 2018; Llovet et al., 2018, 2021; Long et al., 2019b, a; Yang et al., 2019; Craig et al., 2020). Thus, there is a critical need to stratify HCC patients accurately on the basis of heterogeneity and provide precise treatment strategies.

In this study, we analyzed CNAs in tumor cells using CopyKat and classified them into two major subclones, wherein subclone 1 had a higher CNA burden than that of subclone 2. Then, we defined the cells in hepatocyte-like state as the root and cells in high-plasticity state as the end, and reconstructed the tumor evolution trajectory with the help of scRNA-seq data. Consistent with previous studies of other tumors (Zhang L. et al., 2019; Marjanovic et al., 2020), GSVA analysis revealed that along the trajectory, cells gradually lost their intrinsic characteristics and transformed into a high plasticity state. Based on this evolutionary trajectory, we further constructed a 30-gene prognostic model. C-index and multivariate analysis confirmed that compared with the other three existing prognostic models (Bidkhori et al., 2018; Chaudhary et al., 2018; Liang et al., 2020; Sun et al., 2020; Zhang et al., 2020; Deng et al., 2021), this model possessed high predictive efficacy and accuracy. Finally, we also performed GSEA analysis to explore the underlying biological mechanisms of this model.

To investigate the heterogeneity and potential progression trajectory of HCC cells on single-cell resolution, high-quality scRNA-seq data were necessary. Thus, we reviewed associated studies of liver cancer published in recent years to identify the most suitable single-cell dataset (Supplementary Table 3; Zheng et al., 2018; Ma et al., 2019; Zhang Q. et al., 2019; Losic et al., 2020; Sharma et al., 2020; Sun et al., 2021). Two key points need to be considered, namely, the number of tumor cells and the quality of sequencing data. Dissociating tissues is one of the difficulties of single-cell sequencing. Epithelial cells require more stringent dissociation conditions than immune cells and need to be enriched with FACs. However, in early studies, few authors noticed this point, making their results got a large proportion of immune cells and stromal cells instead of tumor cells. This was also the reason why some HCC studies focused on the immune microenvironment (Ma et al., 2019; Ramachandran et al., 2019; Massalha et al., 2020; Sun et al., 2021). To obtain enough cells, we narrowed the range into the data of Sun and Sharma (Sharma et al., 2020; Sun et al., 2021). Regarding the quality of sequencing data, a major factor is the sequencing platform. The plate-based SMART-seq2 full-length method provides in-depth coverage for a smaller number of cells, but the droplet-based 10× Genomics Chromium approach captures cells on a larger scale but with the limitation of inadequate gene coverage. The gene capturing rate of 20 cells by SMART-seq2 was comparable with that of 1,000 cells by 10× (Ding et al., 2019; Zhang Q. et al., 2019). Unfortunately, at least half of the tumor cells from the data of Sharma et al. (2020) based on the 10× platform, could not meet the input threshold of CopyKat, which would introduce a large bias in the downstream analysis (Sharma et al., 2020; Gao et al., 2021). To explore a more refined dynamic change process, we finally chose the data of Sun et al. (2021) for the downstream analysis.

As our model was based on tumor heterogeneity and the trajectory was highly similar to the natural process of tumor evolution, genes included in this model and the underlying biological mechanisms were complicated. Some upregulated genes were found to be associated with cell proliferation and progression. The upregulation of *GPC1* had been reported to be dramatically correlated with the reduced OS time for HCC patients (Wang et al., 2021). *MYCN*, a member of the *Myc* family, was positively correlated with the recurrence of *de novo* HCC (Qin et al., 2018). Furthermore, *EVA1* expression was significantly increased in HCC and was also associated with a poor prognosis and recurrence in these patients. Overexpression of *EVA1* promoted cell growth, invasion, and migration *in vitro*, while knockdown of *EVA1* expression inhibited proliferation and migration *in vitro* (Ni et al., 2020). Some downregulated genes were considered to function as tumor suppressor genes, such as *PPARGC1A*, also known as *PGC-1*α, a master regulator of mitochondrial biogenesis and oxidative phosphorylation. A previous study had reported that low levels of *PPARGC1A* expression were correlated with poor survival, vascular invasion, and large tumor size (Huang et al., 2020; Zuo et al., 2021). *PRICKLE1* has been reported to be a negative regulator of the Wnt/beta-catenin signaling pathway and is a putative tumor suppressor gene in HCC (Chan et al., 2006).

Our study has some limitations. First, RNA-Seq detected more of the content specific to Affymetrix and Illumina microarrays than either of the microarray platforms on the same samples, and many of the feature genes included in our analysis were not detected on the microarray platform (Zhao et al., 2014). Thus, we only used cohorts based on RNA-seq platform. Second, our retrospective findings need to be further validated in prospective research, and complex mechanisms involved in the progression of liver cancer cells still need to be further explored.

**FIGURE 8:**
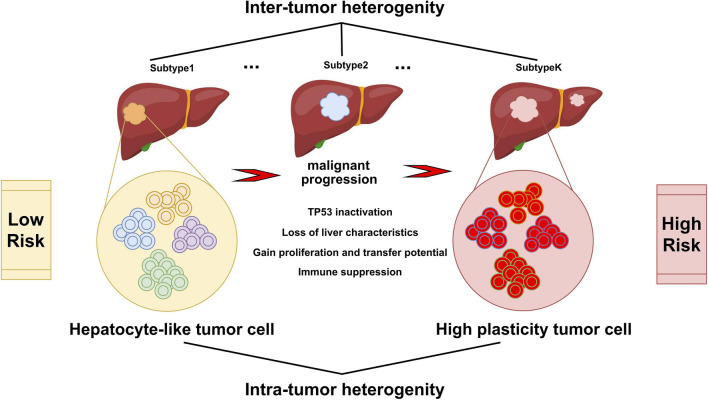
Varied malignant cell subgroups contribute to the inter-tumor and intra-tumor heterogeneity of HCC. Hepatocyte-like tumor cells could progress to high plasticity tumor cells, accompanied by the inactivation of tumor suppressor pathways such as TP53, the disappearance of the inherent characteristics of hepatocytes, the enhancement of proliferation, invasion and metastasis ability, and the appearance of immune suppression.

In conclusion, this study integrated the scRNA-seq data and bulk multi-omics data to reconstruct the tumor evolution trajectory and establish a novel prognostic model to clarify different risk groups of HCC, which might help in clinical decision making for individual treatment and improve patient outcomes (Figure 8).

## Data Availability Statement

The datasets presented in this study can be found in online repositories. The names of the repositories and accession numbers can be found in section “Materials and Methods.”

## Author Contributions

HW, SY, and ZY contributed to conception and design of the study. SY, HW, and QC performed the statistical analysis. HW wrote the first draft of the manuscript. DM, LY, XZ, and JZ wrote sections of the manuscript. All authors contributed to manuscript revision, read, and approved the submitted version.

## Conflict of Interest

The authors declare that the research was conducted in the absence of any commercial or financial relationships that could be construed as a potential conflict of interest.

## Publisher’s Note

All claims expressed in this article are solely those of the authors and do not necessarily represent those of their affiliated organizations, or those of the publisher, the editors and the reviewers. Any product that may be evaluated in this article, or claim that may be made by its manufacturer, is not guaranteed or endorsed by the publisher.

## Footnotes

1
https://xenabrowser.net/

2
https://dcc.icgc.org/projects/LIRI-JP

3
https://portal.gdc.cancer.gov/

4
http://gepia2.cancer-pku.cn/#index

5
https://www.r-project.org/
