# Supplementary material for: The Prognostic Model Based on Tumor Cell Evolution Trajectory Reveals a Different Risk Group of Hepatocellular Carcinoma
Source: Front Cell Dev Biol. 2021 Sep 29;9:737723. doi: 10.3389/fcell.2021.737723 (PMC8511531; doi:10.3389/fcell.2021.737723)
Supplement: Supplementary file 3 [file Data_Sheet_3.PDF]

Supplement Table 3：Datasets review

Data set	GEO Accession	scRNA-seq Method	Total cell number	Tissues	Information
Bojan L et al.(2020), Nat Commun	GSE112271	3′ sequencing and barcoding (10x Genomics)	38553	HCC	2 donors
Ankur S et al.(2020), Cell	GSE156337	3′ sequencing and barcoding (10x Genomics)	73,589	HCC&adjacent	12398 tumor cells from 14 donors (mean number of Features = 659)
Zhang et al.(2019), Cell	HRA000069	3′ sequencing and barcoding (10x Genomics)/Full length (SMART-seq2)	66,187 (10x)<br>11,134(SMART-seq2)	HCC/adjacent/LNs/blood/ascites	High quality CD45+ Immune cells
Ma et al., (2019) Cancer Cell	GSE125449	3′ sequencing and barcoding (10x Genomics)	5, 115	HCC	702 tumor cells from 8 tumors
Sun et al. (2020) cell	CNSA: CNP0000650	Full length (SMART-seq2)	17, 000	HCC	2737 tumor cells from 18 donors (mean number of Features = 7540)
